# Primary chronic osteomyelitis of the jaw – a descriptive study of the condition and its occurrence in Sweden between 2012 and 2018

**DOI:** 10.2340/aos.v83.41989

**Published:** 2024-09-25

**Authors:** Caroline Robertsson, Carina Cardemil

**Affiliations:** a Department of Oral and Maxillofacial Surgery and Jaw Orthopedics, Karolinska University Hospital, Stockholm, Sweden; b Department of Molecular Medicine and Surgery, Karolinska Institutet, Stockholm, Sweden; c Department of Biomaterials, Institute of Clinical Sciences, Sahlgrenska Academy at University of Gothenburg, Gothenburg, Sweden

**Keywords:** Primary chronic osteomyelitis, chronic nonbacterial osteomyelitis, diffuse sclerosing osteomyelitis, mandible

## Abstract

**Purpose:**

Primary chronic osteomyelitis (PCO) of the jaw is a non-infectious, inflammatory state of the jawbone of unknown etiology.

This study aimed to investigate the occurrence of PCO in Sweden between the years 2012 and 2018, the characteristics of the condition, treatment methods, and outcomes.

**Material and methods:**

The search for patients with PCO in Sweden 2012–2018 was performed at 24 oral and maxillofacial surgery units in Sweden.

**Results:**

During this 6-year period, 17 patients were identified as diagnosed with PCO in Sweden. The mean age was 10.6 years at diagnosis, and the female:male ratio was 4:1.

**Conclusion:**

We conclude that PCO is a very rare disease in Sweden, and that standardized, well-defined criteria are necessary to calculate incidence rates but also to increase knowledge about etiology, clinical characteristics, and treatment outcomes in rare conditions such as PCO.

## Introduction

Osteomyelitis is an inflammatory disease of the bone and bone marrow, which may be caused by bacteria, previous surgical intervention, or other trauma to the bone [1]. The characterization of osteomyelitis in the jaw in the literature is highly variable, and many different types of osteomyelitis have been described, differing with respect to localization, prevalence, symptoms, etiology, and progression of the disease [2]. As there are a large number of different classifications of osteomyelitis of the jaw and the nomenclature is still debated, this presents a significant challenge for the clinician when searching for information to select the appropriate treatment [3]. In 2008, Baltensperger presented the ‘Zurich classification system’, and today, this is the most used classification where primary chronic osteomyelitis (PCO) is described as lacking an external cause, whereas secondary chronic osteomyelitis (SCO) is believed to be caused by, for example, bacterial contamination, i.e. previous trauma, dental infection, wounds, or other infections [2]. When PCO affects the other parts of the skeleton, chronic non-bacterial osteomyelitis (CNO) is the most often used term in the literature [4]. A more severe form of the condition is chronic recurrent multifocal osteomyelitis (CRMO), which may affect several skeletal parts simultaneously [5]. Another term often used in the literature is ‘diffuse sclerosing osteomyelitis’ (DSO), which refers to the same condition as PCO and CNO [6, 7].

**Figure 1:**
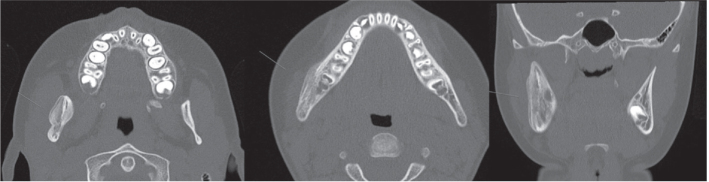
Bone apposition on the right body and ramus of the mandible as well as sclerosis combined with areas of osteolysis and periosteal thickening.

This paper focuses on PCO of the jaw where the patients present with recurrent periods of local swelling, trismus, and pain with no signs of infection. Radiology may show thickening of the periosteum and increased bone width of the jaw, and the recommended imaging technologies include computed tomography (CT), magnetic resonance imaging (MRI), positron emission tomography (PET), and bone scintigraphy [8]. CT is considered as the preferred option as it presents a detailed view of the skeleton and facilitates identifying or ruling out external causes of the osteomyelitis. It is also fast, easily accessible, and has a low cost. A whole-body scintigraphy can be valuable when identifying other affected locations in the body [8]. Biopsy with a bur or osteotome of the affected site is performed to confirm diagnosis but also to rule out neoplasia. When confirming the diagnosis histologically, a highly vascularized fibrous stroma, irregularly distributed osteocytes, and an increased number of osteoblasts (indicative of rapid bone formation) can be observed together with a lack of necrotic cells or sequestration [2] (Figure 1).

Bone culture harvest may also be considered, especially if the etiology is unclear; however, as performing a bone culture in the mandible without contamination from oral bacteria can be challenging, using an extraoral entry is the preferred route [9].

The diagnosis is set after a joint assessment of the clinical and radiological appearance and histopathological results [2]. Today, the first line of treatment usually consists of anti-inflammatory medication (NSAID or steroids) [2,10]. Followed by antiresorptive drugs if little or no effect from NSAID or steroids [7] and finally surgical decortication or resection if the osteomyelitis is resistant to treatment with medication [11, 12]. Up until now, reports in the literature on PCO are almost exclusively case reports and case series [2, 10, 11, 13–17]. In 2020, Andreasen et al. conducted the first systematic clinical trial according to the Declaration of Helsinki, and although being a pilot study, the use of intravenous pamidronate showed positive results, however, non-significant [18].

Compilations of retrospective analyses in different areas or hospitals indicate the rareness of the disease [2, 19], and to our knowledge, this is the first retrospective national compilation of PCO cases. The aim of this report is to describe and characterize the condition after collecting information on all cases registered in Sweden during a 6-year period. Although Sweden is a small country, with only approximately 10 million inhabitants, this study aims to broaden the knowledge of PCO among professionals who encounter and treat these patients.

## Method

The study was designed as a retrospective study analyzing patient charts, aiming to register all cases of PCO diagnosed in Sweden 2012–2018 to study the prevalence and characteristics of the disease. This project was approved by the Swedish Ethical Review Authority, DNR 2018/2184-31.

**Figure 2:**
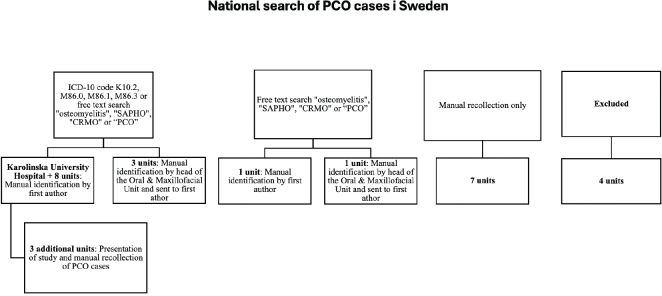
Flow chart of search method.

All 28 oral and maxillofacial surgery (OMS) units in Sweden were invited to participate and contribute to the search. As PCO does not have a specific International Statistical Classification of Diseases-10 (ICD-10) code, a manual search was necessary at all units. Since the enforcement of the General Data Protection Regulation (GDPR) in 2018, a higher standard when handling personal data have been applied, including stricter regulations regarding extraction of data. Separate search methods had to be applied due to different qualities of the patient chart systems used in the country and the laws regulating patient confidentiality in Sweden but also the varying interpretations of the regulations in different council regions of the country (Figure 2).

All patients treated in an OMS unit in Sweden, including associated specialist dental clinics or associated ear, nose, and throat departments, during 2012–2018 that clinically and radiologically matched the diagnosis *PCO of the jaw,* without an observed outer cause, were included in the study.

The clinical signs that were assessed as indicators of osteomyelitis were swelling, trismus, and pain with no signs of infection, while the radiological signs were increased bone width with irregular bone matrix, thickening of periosteum, and absence of infected teeth, implants, or trauma site in proximity to the anatomical site affected by osteomyelitis.

The following patients were excluded from the study: (1) Patients with an incomplete patient chart, where it was not possible to objectively or through the anamnesis assess whether the condition was bacterial or not. (2) Patients where the diagnosis *PCO of the jaw* was set while the data in the chart suggest a bacterial cause. (3) Patients who had received radiation therapy toward the head and neck area or had previously been treated with antiresorptive drugs.

The first search was performed at Karolinska University Hospital, where a data search of the patient chart system covering all patients treated between Jan 1, 2012 and Dec 31, 2018 was executed. All patients with the ICD-10 code K10.2, M86.0, M86.1, M86.3 or with a patient chart that included one of the words ‘osteomyelitis’, ‘SAPHO’, ‘CRMO’, or ‘PCO’ during this time were selected out, whereafter the first author searched these patient charts to identify patients eligible for inclusion in the study. The same method was applied at eight other OMS units.

In three other units, the same search method was used; however, here the head of the OMS unit searched the patient charts in order to identify possible cases of PCO, after which these charts were sent to the first author for the possible inclusion.

In yet two other units where ICD codes are not used in the charts, a free text data search of the patient chart system of all patients between Jan 1, 2012 and Dec 31, 2018 was performed. In the first of these two clinics, the first author screened the patient charts assessed as possibly containing cases of PCO. In the second clinic, identification of possible cases of PCO was made by the head of the clinic after which the charts were sent to the first author for possible inclusion in the study.

In three additional clinics, a data search as described for the search at Karolinska University Hospital was performed, followed by the first author manually studying the charts to identify patients matching the inclusion criteria. Finally, a presentation of the study was made to all surgeons at the clinics where they were asked if they recollected any cases of PCO that could be considered for inclusion in the study.

In seven units, the head of departments took the decision that a search was not necessary or not possible and enquired the surgeons for manual identification.

Hence, to get information from the latter clinics, the authors had to rely on the patient observance and memory of the surgeons.

One clinic was contacted but directly stated that they do not treat patients with PCO.

One single council region in Sweden, where three clinics are located, decided not to participate in this study, stating that resources could not be set aside for this survey. Therefore, 86.4% of the population in the country of Sweden is covered in this study.

## Results

### Age, characteristics

A total of 17 cases fulfilled the inclusion criteria in the Swedish national search of patients with a confirmed diagnosis of PCO during the years 2012–2018. The mean age at symptom debut was 9.9 years, while the mean age at diagnosis was 10.6 years, resulting in an average time between symptom debut and diagnosis of 8 months. Thirteen out of 17 patients were female (76%), while four were male (24%). Regular dentists, pediatric dental clinics, general practitioners, and ear-nose-throat specialists referred the patients to the OMS units. All but two patients had received the ICD-10 code K10.2, while the other two were diagnosed with the M86.9 code. No abnormal tooth growth was registered in any of the patients, who all had normal exfoliation of teeth for their age.

### Medical history

The major part of the patients included in the study had previously been healthy at the time of diagnosis (82.4%), except for three patients (17.6%). One patient had ongoing allergy desensitization treatment due to a history of severe allergies and was also treated with cetirizine, an antihistamine. One female patient had an early onset of puberty and received triptorelin (a gonadotropin-releasing hormone agonist) injections to pause entry into puberty. Another patient had psoriasis with no active treatment.

Two (one male and one female) out of 17 patients reported a growth spurt at the time when symptoms of PCO first started to appear; however, the other 15 did not report the contrary.

### Symptoms and findings

**Figure 3:**
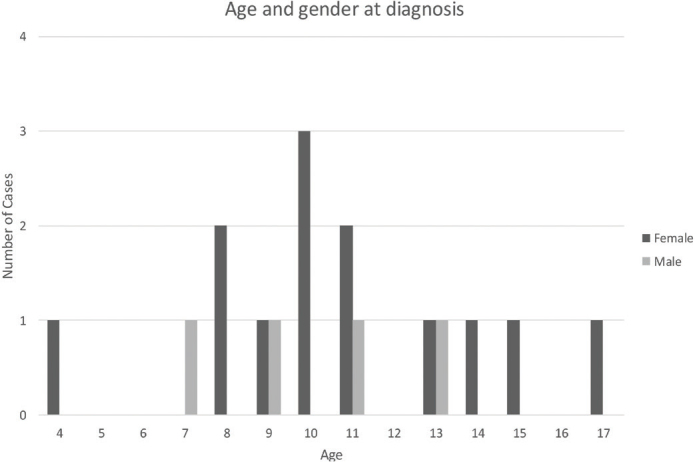
Distribution of age and gender at time of diagnosis.

**Figure 4:**
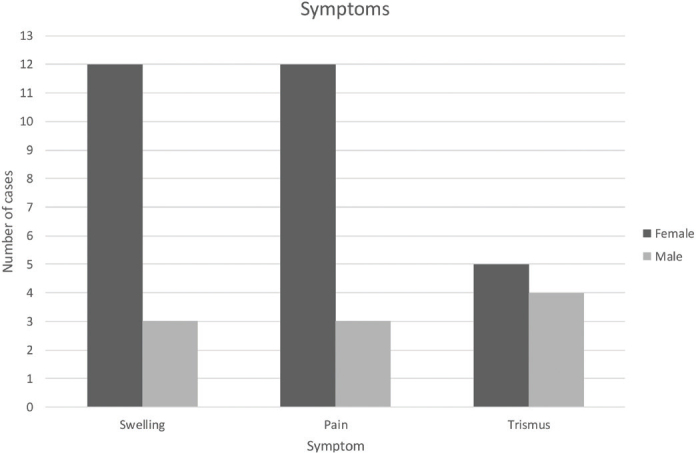
Distribution of reported symptoms.

Fourteen out of 17 charts described complete blood count with results showing all cells within normal levels and an elevated C-reactive protein in all patients. One out of 17 patients had confirmed skeletal multifocal lesions located in the right acromion, and two patients had knee pain but no confirmed bone pathology. Fifteen patients reported swelling, trismus, and pain on the affected side of the jaw. Two patients reported limited mouth opening as the only symptom (Figures 3 and 4).

### Diagnosis and treatment

Thirteen patients were subject to biopsy to confirm the PCO diagnosis whereof three patients had a wisdom tooth removed in the affected area at the same occasion and one patient underwent surgical decortication. The other four patients received their diagnosis by assessment of clinical and radiological appearance. All 17 patients underwent radiology to confirm the diagnosis, and all were treated with NSAIDs in different forms (i.e. naproxen, ibuprofen, diclofenac) for the duration of the symptoms. Outcomes vary from patients being completely relieved of their symptoms to no effect of the medication at all. Ten out of 17 patients were prescribed antibiotics (Phenoxymethylpenicillin or Clindamycin) with a limited or no effect at all. Six out of 17 patients received treatment with corticosteroids. Additional treatments used were methotrexate, folic acid, pamidronate, tumor necrosis factor-alpha (TNF-α) inhibitor, and hyperbaric oxygen treatment.

The six patients who either received TNF-α inhibitor, triptorelin, melatonin, or pamidronate all reported a significant improvement after treatment in terms of pain and swelling.

### Patient follow-up

Twelve out of 17 patients were without symptoms at the end of this study. Eleven patients were under continued check-ups at OMS units in Sweden.

## Discussion

During the recent two decades, a shift has occurred in the view of PCO, and it is no longer regarded as a condition caused by bacterial infection. PCO is still a poorly understood disorder and is often misdiagnosed as bacterial osteomyelitis. The diffuse onset of symptoms in PCO as well as the rarity of the disease often leads to difficulties in setting the diagnosis and subsequently to treat this patient group [2, 20]. This concurs well with the findings in this study, where the mean time from symptom debut to diagnosis was 8 months.

As this is such an uncommon disease, there are no prospective cohort studies as of now, due to the difficulty of gathering enough cases; therefore the greater part of publications are case reports and case series. The main finding of this study is the rarity of the disease in Sweden, which thus confirms that larger compilations are needed to increase the knowledge. This study also indicates that primarily individuals in late childhood are affected by PCO; however, the material is too limited to draw such a conclusion. It concurs with earlier findings in other studies, where most of the presented cases of PCO are of children and adolescents [1, 2, 6, 10, 14–17, 21–23], including the 22 cases of PCO presented in the study by Gaal et al. [19]. This raises the question of whether there are specific mechanisms in the growing individual that increase the risk of developing PCO. The distribution of the condition between the sexes differs in the literature; however, the findings in this study correspond with numerous case reports that there is a female predisposition [2, 8, 9, 15–17, 24]. Contrarily, Gaal et al. observed the opposite in their study, describing a female-to-male ratio of 1–3 [19].

Døving et al. and Clover et al. have reported of two healthy females who during the first trimester of pregnancy developed PCO of the jaw [25, 26]. Several hormonal changes occur during pregnancy, and levels of estradiol increase significantly, which bears some similarities to the hormonal changes of females entering puberty.

Remodeling of the skeleton is a constant process dependent on the bone-resorbing osteoclasts and the bone-forming osteoblasts. Together growth factors, hormones, and mechanical stress will stimulate both trabecular and cortical bone formation but in slightly different ways [27]. The entrance into puberty with a spike of sex hormones is crucial to the growth but also to the increase in strength of the skeleton [28]. Hence, it would be possible that a rapid increase in sex hormones can contribute to the development of PCO. However, only increased bone formation cannot explain the development of PCO. In one case report, Rasmussen et al. show that bone metabolism factors such as plasma alkaline phosphatase, plasma CTX, and plasma osteocalcin were increased in a patient with PCO of the jaw [29], and similar results are shown in a case series of 14 patients with chronic nonbacterial osteomyelitis in various anatomic locations [30]. These indicators of bone remodeling may reflect mechanisms causing PCO or may be an effect of the condition. Further investigations are required to determine whether PCO primarily is a disease of imbalance in bone metabolism with a secondary inflammation or contrariwise, with a pathological process initiated by inflammation.

In this study, we found that all 17 patients initially received NSAIDs, 13 of these received NSAIDs in combination with other medications. In the latter group of 13 patients who had used other drugs concomitant to NSAID, five still had symptoms, while the four patients who received NSAID only were free of symptoms at the end of this study. It correlates well with the current recommendations that NSAIDs are the first line of treatment for PCO [2].

In a retrospective study of 22 patients treated for PCO, it was shown that treatment with both TNF-α inhibitor and bisphosphonate medication was superior to NSAIDs alone. To achieve a situation where the patient was relieved from all symptoms, a shorter treatment time was needed when TNF-α inhibitor and bisphosphonates were administered together [19]. This correlates well with the findings of this study where the six patients who had received TNF-α inhibitor, triptorelin, or pamidronate all reported a significant improvement after treatment. These six patients all received medication with a drug affecting bone metabolism, suggesting that alterations in bone remodeling may have a positive effect on PCO.

The inflammatory bone disorder CRMO, described as an autoimmune disease, can present with multiple bones of the body affected by osteomyelitis without a known bacterial or traumatic incident in the patient history [31]. It is not known if the mechanisms involved in the development of CRMO and PCO have any similarities although it could be hypothesized, as the two conditions appear to affect the same age group. In our study, only one out of 17 patients had a confirmed skeletal pathology outside of the jaws, in this case in the acromion; however, this finding well illustrates why there is recommendation to perform a whole-body scintigraphy in order to rule out lesions in other bones. The etiologies of both these conditions, CRMO and PCO are only partly understood, and today different types of treatments are still suggested as there is a lack of consensus on treatment guidelines [23, 32].

The de-centralized health care system in Sweden, divided into six health care regions and 21 region councils, all with their own regional jurisdictional regulations, has several drawbacks when performing research of a rare disease entity with a limited number of patients, one of them being the usage of different patient chart systems in the separate regions as well as within the regions. There are also different ways of how to register diagnoses as well as multiple ways of searching the databases and in this specific case, as PCO does not have a unique ICD code, a manual study of the charts was required.

In several regions, the authors had to rely on colleagues being able to identify the cases treated at their centers; however, as PCO is such an unusual disease, the surgeons most often do remember these patients. Another aggravating factor when collecting the data from the different regions was the COVID-19 pandemic due to travel bans.

Patients with PCO are treated at different health care facilities in Sweden, which further complicated the search of cases with this diagnosis. Most of the patients eventually get their treatment at an OMS unit, wherefore searches were performed at such clinics; however, PCO patients are occasionally referred to an Ear Nose Throat department, to pediatric rheumatology or endocrinology departments and to pedodontic clinics, entities that are not covered by this study.

There is also a possibility that the surgeon has a preconception of an infectious origin when setting the diagnosis and searches for a dental and therefore a bacterial cause although there is none present. This path of investigation was observed in many patient charts and may be explained by the fairly recent shift in how the etiology of PCO is viewed. A tendency to pursuit a microbiological cause is also evident when studying the charts in this study, as there are examples of when antibiotics are still being prescribed even though the patient lacks signs of infection (e.g. fever, high white blood cell count, pus, or positive bacterial culture). This illustrates the challenges in reaching a correct diagnosis, which could result in a delay of appropriate treatment for the PCO patient.

Another limitation of this study is that medical history and the commencement of the symptoms were not always clearly described in all the charts, especially in older patients where dental treatment often had been performed, wherefore SCO could not be excluded.

Despite the difficulties encountered in the compilation of the cases reported in this study, the results may raise awareness of the significance of including a good medical history in the patient chart for future patient management and to facilitate research on rare diseases. The study also highlights the importance of an increased collaboration between dental and medical specialties as PCO of the jaw and CNO/CRMO are conditions that may possibly derive from similar mechanisms.

## Conclusion

We conclude that PCO is a very uncommon condition in Sweden and that finding an effective and safe treatment is still difficult for the specialists involved in the care of this patient group. Another important finding is that performing a national survey of PCO is challenging, which emphasizes the need of a consensus on how to set the diagnosis of PCO of the jaws, as well as establishing an international diagnosis code for the condition. This would enable larger studies to validate the results indicated in this study and also facilitate further investigations on the mechanisms involved in the etiology of PCO of the jaws.
